# Pharmacokinetic Profile of Kaurenoic Acid after Oral Administration of Araliae Continentalis Radix Extract Powder to Humans

**DOI:** 10.3390/pharmaceutics10040253

**Published:** 2018-12-01

**Authors:** Eun-Jeong Choi, Go-Wun Choi, Seung-Jeong Yang, Yong-Bok Lee, Hea-Young Cho

**Affiliations:** 1 College of Pharmacy, CHA University, 335 Pangyo-ro, Bundang-gu, Seongnam-si, Gyeonggi-do 13488, Korea; choiej5048@gmail.com (E.-J.C.); gwchoi153@gmail.com (G.-W.C.); 2 Department of Korean Obestetrics and Gynecology, Dong-shin University, 141, Wolsan-ro, Nam-gu, Gwangju 61619, Korea; cigipus@hanmail.net; 3 College of Pharmacy, Chonnam National University, 77 Yongbong-ro, Buk-Gu, Gwangju 61186, Korea; leeyb@chonnam.ac.kr

**Keywords:** *Aralia continentalis* Kitagawa, kaurenoic acid, pharmacokinetics, Caco-2 cell, clinical trials

## Abstract

The objective of this study was to characterize pharmacokinetics (PKs) of kaurenoic acid (KAU) after administration of the clinical usual dose of Araliae Continentalis Radix extract powder to Korean subjects for the first time and evaluate the mechanism of its absorption *in vitro*. A simple, sensitive, and selective analytical method was developed for the detection of KAU in human plasma. Concentrations of KAU were quantified by ultra-performance liquid chromatography tandem mass spectrometry after simple liquid–liquid extraction. This pharmacokinetic model of KAU was best described by a two-compartment model with first-order absorption. To identify efflux transporters involved in the absorption of KAU, a Caco-2 monolayer model was used. Estimated PK parameters were: systemic clearance, 23.89 L/h; inter-compartmental clearance, 15.55 L/h; rate constant for absorption, 1.72 h^−1^; volume of distribution of the central compartment, 24.44 L; and volume of distribution of the peripheral compartment, 64.05 L. Results from Caco-2 bidirectional transport study suggested that KAU was a potential substrate of efflux transporters. In summary, PKs of KAU were successfully characterized after administration of a usual dose of Araliae continentalis Radix extract powder in human with the newly developed bioanalytical method and the mechanism of absorption of KAU was identified clearly.

## 1. Introduction

*Aralia continentalis* Kitagawa has been extensively cultivated in Asia, Siberia, China, and Korea. Its dried root, Araliae continentalis Radix, is a perennial herb that has been widely used for therapeutic uses such as rheumatism, lumbago, and lameness [1], and it has antinociceptive [2], antidementia [3], antioxidant [4], anticancer [5], and anti-inflammatory activities [6]. In Korea, Araliae continentalis Radix extract powder has been approved by the Ministry of Food and Drug Safety (MFDS). It is commercially available. It is usually administered 0.72–1.09 g per oral three times daily to patients with pain and convulsion.

In phytochemical investigations, various diterpenes, flavonoids, saponins, and essential oils have been isolated from roots and leaves of this plant [7]. Kaurenoic acid (KAU) is one of the representative diterpenes isolated from *Aralia continentalis*. It possesses various biological activities, including anticonvulsant [8], tumor suppression [9], leukemia apoptosis [10], and anti-inflammatory. It also have antibacterial [11], antifungal [12], anti-leishmanial [13], anti-plasmodial [14], anti-syphiliticus [15], and anti-cariogenic [16] activities. Isolation of KAU from other herbs such as *Mikania glomerata* [17], *Copaifera langsdorffii* [18], *Sphagneticola trilobata* [19], *Pseudognaphalium vira vira* [12], and several *Annonaceae families* [8,20,21,22] has also been reported. In spite of these therapeutic uses, KAU has to be used carefully because of its toxic effect. Costa-Lotufo et al. [18] has demonstrated its cytotoxic and embryotoxic effects by observing embryonic cell of sea urchins and hemolysis of mouse and human erythrocytes. Cavalcanti et al. [20] has identified its genotoxic and mutagenic effects in human leukocytes, yeast, and mice tissue cells.

Pharmacokinetic (PK) studies for safety evaluation could provide information about the dose-exposure relationship which is of primary concern in the drug development process and clinical use [23]. Herbal medicines have various and variable components when these phytochemicals are introduced into human body [24]. Therefore, robust and sensitive bioanalytical methods are essentially required prior to assessment of the safety and efficacy of herbal medicine.

During the past decades, bioanalytical methods for KAU have not been reported. PK study of KAU has not been reported at all. Recently, Gasparetto et al. [25] has reported an analytical method of KAU with coumarin metabolites in human plasma after oral administration of guaco syrup. They developed and validated the analytical method of these compounds using spiked calibration and quality control (QC) samples. However, they could not detect KAU in human plasma after oral administration of guaco syrup [25]. Matos et al. [26] has reported PK profile of KAU in rats after intravenous and oral administration. Although they developed a two-compartment PK model, they could not evaluate the bioavailability of KAU because of insufficient low limit of quantification (LLOQ, 5 ng/mL) for quantification of KAU in rat plasma after oral administration of KAU at 50 mg/kg. Due to challenging aspects of quantitative analysis, the evaluation of PKs for KAU has not been reported in clinical study yet.

To have a better understanding of PK behavior of KAU, transport study is necessary to clarify its absorption mechanism. However, transport assay of KAU has not been published yet. Three major ATP-binding cassette transporters including P-glycoprotein, multidrug resistance protein [27], and breast cancer resistance protein [27] play important roles in the efflux of xenobiotics and endogenous substrates from cells [27]. Caco-2 cell expressing three efflux transporters is a cell line that is widely used as a standard screening tool to evaluate the absorption mechanism of transport of drug candidates [28]. Thus, Caco-2 monolayer model was chosen in this study.

The objective of this study was to evaluate PKs of KAU after clinical usual dose of Arailae continentalis Radix extract powder orally administered to healthy Korean subjects and identified the mechanism of absorption of KAU to interpret the behavior of KAU in human body. In addition, a simple, sensitive, and selective bioanalytical method using UPLC-MS/MS was developed to determination of KAU in human plasma.

## 2. Materials and Methods

### 2.1. Chemicals and Reagents

**Figure 1:**
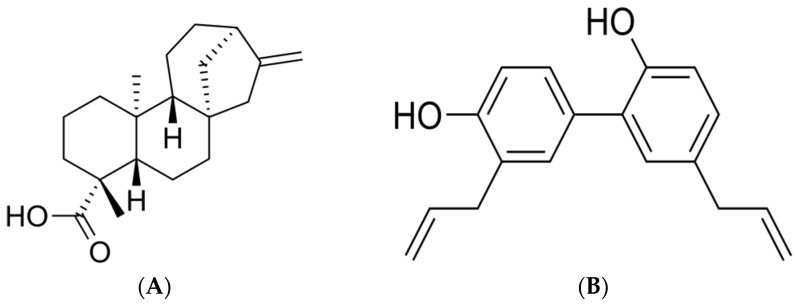
Structures of the investigated compound and internal standard (IS). (**A**) Kaurenoic acid (KAU), and (**B**) IS.

The reference standard (RS) of KAU (purity > 98.6%) and honokiol used as an internal standard (IS) (Figure 1) were obtained from the Ministry of Food and Drug Safety (Cheongju-si, Chungcheongbuk-do, Korea). Methanol, acetonitrile, and methyl-*tert*-butyl ether were purchased from J.T. Baker (Phillipsburg, NJ, USA). Ammonium acetate, formic acid, and trifluoroacetic acid were purchased from Sigma-Aldrich (St. Louis, MO, USA). HPLC grade water (18.2 MΩ) was obtained using an Elga Purelab option-Q system (Elga Labwater, Marlow, UK). It was used throughout this study. Other chemicals were of HPLC grade or the highest quality available.

### 2.2. Preparation of Herbal Extract

Araliae continentalis Radix extract powder was purchased from Hankook Shin Yak Corp (Nonsan-si, Chungcheongnam-do, Korea). It was manufactured according to the Korean pharmacopoeia codex. One kg of the root of *Aralia continentalis* Kitagawa (Araliae continentalis Radix) was extracted with 80–100 °C water (10 L) for 2–3 h. To gain dried extract powder, the extract solution was filtered and evaporated under vacuum. Then 100 mg of dried extract powder was physically mixed with 168 mg of lactose.

### 2.3. Content of KAU 

Content of KAU in Araliae continentalis Radix extract powder was determined using assay test for Araliae continentalis Radix extract powder described in the Korean Pharmacopeia (Ministry of Food and Drug Safety, 2012). Briefly, 1.0054 g of extract powder was accurately weighed and 50 mL of 100% ethanol was added for extraction by sonication for 30 min. The extract was then filtered by filter paper. To the residual extract powder, 50 mL of 100% ethanol was added followed by extraction under ultrasound sonication for 30 min. The mixture was then repeatedly filtered. Filtered extract was concentrated under vacuum condensation. It was then resuspended in exactly 50 mL of 100% ethanol. This extract was filtered using a 0.45 µm syringe filter. Separately, 1.0 mg of KAU of RS was weighed and dissolved in 100% ethanol to make 1000 µg/mL. The resulting solution was used as standard solution. Each 20 µL of the test solution and the standard solution were injected directly to HPLC. KAU content was analyzed by HPLC using Waters Nova-Pak^®^, C_18_ column (3.9 mm × 150 mm, 4 µm particle size, Waters, Milford, MA, USA). Separation was carried out with gradient elution procedure using 0.1% trifluoroacetic acid in water (mobile phase A) and acetonitrile (mobile phase B). The composition of mobile phases used was as follows: 0–5 min, 10% of B; 5–15 min, 10–60% of B; 15–20 min, 60% of B; 20–25 min, 60–10% of B; and 25–30 min, 10% of B. The wavelength for UV detection was set at 208 nm and the flow rate was set at 1.0 mL/min. Peak areas of KAU of the test solution (A_T_) and the standard solution (A_S_) were determined. Content analysis was performed in triplicates. The amount of KAU is reported as mean ± SD.

Amount (mg) of KAU = amount (mg) of KAU RS × (AT/AS)(1)

### 2.4. Quantification of KAU in Biological Samples

Quantitative analysis of KAU in human plasma was conducted with the newly developed bioanalytical UPLC-MS/MS method. This bioanalytical method was optimized via lots of trials for various mobile phase conditions and sample preparation procedure. Each 200 µL of plasma sample was prepared by adding 10 µL of the IS solution (10 ng/mL of honokiol in 50% methanol) to reduce error in sample preparation procedures. Samples added with IS solution were simply extracted by liquid–liquid extraction (LLE) [28] using methyl-*tert*-butyl ether. Then 1000 µL of methyl-*tert*-butyl ether was added to separate the analyte from impurities. The mixture was then vortexed for 3 min and centrifuged at 10,000 × *g* for 5 min. Then 900 µL of the supernatant organic layer which was separated from aqueous layer was transferred to a clean microtube and dried under a nitrogen steam at 50 °C in a centrifugal evaporator. The dried matter was reconstituted with 100 µL of 50% acetonitrile in water and vortexed for 1 min. After centrifugation at 10,000 × *g* for 5 min, 10 µL of the aliquot was injected into the UPLC-MS/MS system. Liquid chromatography was performed on an Acquity™ UPLC^®^ system (Waters Corp., Milford, MA, USA) coupled to a Mass Spectrometer (Xevo™ TQ-S, Waters Corp., Milford, MA, USA). Each aliquot was injected into a Kinetex C_18_ column (2.1 mm × 50 mm, 1.7 µm particle size, Phenomenex, Torrance, CA, USA) at a column temperature of 40 ± 0.5 °C. Separation was carried out by gradient elution procedure using 5 mM ammonium acetate in water (mobile phase A) and acetonitrile (mobile phase B). The ratio of both mobile phases was as follows: 0–1 min, 50% B; 1–3 min, 80% B; and 3–4 min, 50% B. The mass spectrometer was operated with electrospray ionization interface in negative ion mode. Multiple reaction monitoring (MRM) transitions such as m/z 301.2→301.2 and 265.2→224.2 were used for KAU and IS, respectively. The following parameters were used: capillary voltage, 2.8 kV; ion source temperature, 150 °C; and desolvation temperature, 350 °C. Nitrogen was used as the cone and desolvation gas at a flow rate of 150 and 550 L/h, respectively. The optimized collision energy was 30 eV for KAU and 22 eV for IS. Cone voltages of KAU and IS were 30 V. Argon gas was used at a pressure of approximately 4.2 × 10^−3^ mbar. Data acquisition and analysis were achieved using Masslynx 4.1 software (Waters Corp., Milford, MA, USA). The developed UPLC-MS/MS method was validated for selectivity, accuracy, precision, recovery, calibration curve, sensitivity, and stability (at room temperature and −80 °C, after freeze and thaw cycles, and at 10 °C in an autosampler after the preparation procedure) according to the Guidance for Industry: Bioanalytical Method Validation established by FDA [29].

### 2.5. Clinical Trials Design

Ten healthy male Korean subjects (21–33 years, 60.4–86.7 kg, and 167–183 cm) were enrolled in an open labeled study for investigator-initiated clinical trial. Our investigator-initiated clinical study is a part of the project of “Pharmacological Standardization of Herbal Preparation” to determine PKs after oral administration of Araliae continentalis Radix extract to healthy male subjects. All subjects participated in this study after providing written informed consent. This study protocol was approved by the Institutional Review Board of Oriental Medicine Hospital of Dongshin University, Suncheon-si, Jeollanam-do, Korea (https://cris.nih.go.kr) (approval number: KCT0001211). This study was carried out according to the revised Declaration of Helsinki for biomedical research involving human subjects and rules of Good Clinical Practice. These subjects were in good health prior to this study as confirmed by physical examination, medical history and laboratory tests, including hemoglobin, hematocrit, red blood cell (RBC), white blood cell (WBC), platelet, albumin, total cholesterol, and triglyceride, hepatic function test, alkaline phosphatase (ALP), alanine transaminase [30], and aspartate transaminase (AST), renal function test, blood urea nitrogen [31], creatinine, and creatinine clearance (CrCL). These subjects were selected if there was no possibility of being sensitive to this medication and if they had no history of illness of hepatic, renal, or cardiovascular system and no history of excessive alcohol intake or other medications. All subjects were fasted for at least 10 h before administration of the extract powder. They continued to fast for 4 h thereafter. They abstained from consumption of alcohol, xanthine-containing food, and xanthine-containing beverage during the study. Each subject received clinical oral dose of Araliae continentalis Radix extract powder (1.0054 g) with 240 mL of spring water. The administered dose is a clinical usual dose of Araliae continentalis Radix extract powder approved MFDS, Korea.

Blood was drawn from the forearm vein before administration and at 0.25, 0.5, 0.75, 1, 2, 3, 4, 6, 8, 10, and 12 h after oral administration. Blood samples were transferred to Vacutainer^®^ (10 mL, Becton, Dickinson and Company, Franklin Lakes, NJ, USA) tubes and immediately centrifuged (10,000 × *g*, 10 min, 4 °C) to obtain plasma samples. After separation of plasma from blood samples, 5 mL aliquots of plasma were transferred into a polyethylene tube and stored at −80 °C until analysis.

### 2.6. Pharmacokinetic Analysis

PK analysis was performed by non-compartmental and compartmental analyses using Phoenix WinNonlin with conventional built in PK models and NLME with naive pooled engine (version 8.0, Pharsight^®^, a Certara™ Company, Princeton, NJ, USA). 

PK parameters were estimated using the following ordinary differential equations:

(2) $$\frac{{\mathrm{dA}}_{a}}{\mathrm{dt}}=-k_{a}\cdot A_{a}\;\left(A_{a}=F\cdot \mathrm{dose}\;\mathrm{at}\;\mathrm{time}=0\right)\;\frac{{\mathrm{dA}}_{1}}{\mathrm{dt}}=A_{a}\cdot k_{a}-\mathrm{CL}\cdot \frac{A_{1}}{V}-{\mathrm{CL}}_{D}\cdot \left(\frac{A_{1}}{V}-\frac{A_{2}}{V_{2}}\right)\;\frac{{\mathrm{dA}}_{2}}{\mathrm{dt}}={\mathrm{CL}}_{D}\cdot \left(\frac{A_{1}}{V}-\frac{A_{2}}{V_{2}}\right)$$

where A_a_ was the amount in the absorption compartment, t was time, k_a_ was the first order absorption rate, dose was the administered amount of KAU per oral, A_1_ was the amount in the central compartment, CL was systemic clearance, V was the volume of distribution of the central compartment, CL_D_ was inter-compartmental clearance, V_2_ was the volume of distribution of the peripheral compartment, and A_2_ was the amount in the peripheral compartment. In this model, CL, CL_D_, V, and V_2_ were apparent parameters that actually included fraction (F) of absorption.

To select the optimal PK model for KAU, goodness of fit via visual inspection, diagnostic plots of weighted residuals and scatter plots of observations versus model predicted plasma concentration, and general diagnostic criteria such as akaike information criteria (AIC), condition number, count of minimization iteration, and twice the negative log likelihood were used.

A proportional error model was used to describe residual errors (ε) of the data that were randomly distributed with a mean of zero and a variance of σ^2^ with the following equation:

(3) $$C_{\mathrm{obs}}=C_{\mathrm{pred}}\times \left(1+\varepsilon \right)$$

where the observed plasma concentration of KAU for individuals (C_obs_) was presented as the model predicted concentration (C_pred_) plus the error value (ε).

Secondary PK parameters such as maximum plasma concentration (C_max_), time to reach the C_max_ (T_max_), area under the concentration-time curve from zero to infinity (AUC_0-∞_), and elimination half-life (t_1/2_) were determined by non-compartmental analysis with data obtained from model predicted time-plasma concentration of KAU. C_max_ and T_max_ were determined by visual observation of the plasma concentration–time curve. AUC_0-∞_ was integrated by linear trapezoidal rule from time zero to the final measured concentration. It was extrapolated from the final measured concentration to infinity.

### 2.7. Cell Culture

Caco-2 cells were obtained from the American Type Culture Collection (Manassas, VA, USA). Cells were cultured on tissue culture flasks in Dulbecco’s modified Eagle medium supplemented with 10% fetal bovine serum (Corning, NY, USA) and 1% penicillin streptomycin (Corning, NY, USA). These cells were maintained in an atmosphere of 5% CO_2_/95% air at 37 °C. The culture medium was refreshed every 3 to 5 days after detaching with trypsin-EDTA. For transport experiments, Caco-2 cells with passage number 49 were seeded into 12-well Transwell plates (1.12 cm^2^ in surface, 0.4 μm in pore size, 12 mm in diameter, Corning Costar Corporation, Cambridge, MA, USA) at a density of 3.5 × 10^5^ cells/insert and cultured for 3 weeks. The integrity of cell monolayer was evaluated prior to transport experiments by measuring transepithelial electrical resistance (TEER). TEER values greater than 200 Ωcm^2^ for Caco-2 cell monolayers were used in the transport experiment.

### 2.8. In Vitro Cytotoxicity Assay

Cytotoxicity of KAU to Caco-2 cells was evaluated using Cell Counting Kit-8 (CCK-8) reagent (Dojindo Molecular Technologies, Inc., Washington, D.C., MD, USA). Briefly, these cells were seeded into 96-well plates at a density of 1.0 × 10^4^ cells/well followed by pre-incubation for 24 h at 37 °C with 5% CO_2_. Then 10 µL of KAU at various concentrations was added into each well. After incubation and exposure for 24 h in the incubator, CCK-8 reagent (10 µL) was added to each well of the 96-well plate. After incubation at 37 °C with 5% CO_2_ for 4 h, absorbance of each well was measured at 450 nm using a 96-well plate reader (SpectraMax i3x, Molecular Devices, LLC, San Jose, CA, USA). Cell viability (%) was calculated based on the measured value relative to the absorbance of cells exposed to the negative control. All data are shown as mean ± standard deviation (SD) (n = 6). Statistical comparisons were made with Student’s *t*-test. *p*-values of less than 0.05 were considered statistically significant.

### 2.9. Bidirectional Transport Assay

Bidirectional transport assay was conducted based on previous reports [32,33] and FDA guidance [34]. Before transport experiments, Caco-2 cell monolayers were washed twice and incubated at 37 °C for 30 min with transport buffer (Hank’s balanced salt solution with 10 mM HEPES, pH 7.4). Then 10 mM stock solution of KAU was prepared in DMSO and diluted with transport buffer to 1, 10, 50, and 100 μM. Each concentration of KAU was added onto either the apical (0.5 mL) or basolateral side (1.5 mL) and plates were shaken gently for 2 h at 37 °C on a plate shaker. After 200 μL sample was taken from the receiver side at 15, 30, 45, 60, 90, and 120 min, the same volume of fresh transport buffer was added as replacement. Each sample was analyzed with established UPLC-MS/MS method to calculate apparent permeability coefficients (P_app_) as shown below:

(4) $$P_{\mathrm{app}}=\frac{\mathrm{dQ}/\mathrm{dt}}{{\mathrm{AC}}_{0}}$$

where dQ/dt (μmol/s) is the cumulative rate transported of KAU on receiver side, A (cm^2^) is the membrane surface area, and C_0_ (μM) is initial concentration in donor compartment.

The efflux ratio was calculated by:

(5) $$\mathrm{Efflux}\;\mathrm{ratio}=\frac{P_{\mathrm{app}\left(\mathrm{BL}\to \mathrm{AP}\right)}}{P_{\mathrm{app}\left(\mathrm{AP}\to \mathrm{BL}\right)}}$$

where P_app(BL→AP)_ was the P_app_ of basolateral to apical side and P_app(AP→BL)_ was the P_app_ of apical to basolateral side.

## 3. Results and Discussion

### 3.1. Content of Kaurenoic Acid

Average content (± SD) of KAU in the Araliae continentalis Radix extract powder was estimated to be 1.1408 ± 0.008 mg/g. For clinical trial, clinical usual dose of 1.0054 g Araliae continentalis Radix extract powder containing 1.147 mg of KAU was orally administrated to humans.

### 3.2. Quantification of KAU

UPLC–MS/MS has been emerging as a powerful analytical technique for determination of analyte in biological samples to improve sensitivity and selectivity. Herein, we developed a simple, selective, and sensitive bioanalytical UPLC-MS/MS method for quantification of KAU after oral administration of Aralia continentalis Radix extract powder to humans. Prior to this study, Gasparetto et al. [25] reported kinetic profiles of Main Guaco metabolites using syrup formulation after 60 mL Guaco syrup containing 8.9 mg of KAU was administered to humans. However, the PK profile of KAU was not identified due to a high LLOQ of 5 ng/mL which was 25 times higher than our LLOQ at 0.2 ng/mL. Matos et al. [26] has also reported the PK profile of KAU in rats after administration of KAU at 50 mg/kg per oral or intravenous route. Considering a normal rat’s weight was 0.25 kg, approximately 12.5 mg of KAU was administered to each rat in their study. However, the PK profile after oral administration was not identified due to the same limitation (i.e., LLOQ of 0.75 μg/mL was too high, which was 3750 time higher than our LLOQ). In contrast, our developed bioanalytical UPLC-MS/MS method was able to successfully quantitate KAU after usual oral administration of Araliae continentalis Radix powder to human.

**Figure 2:**
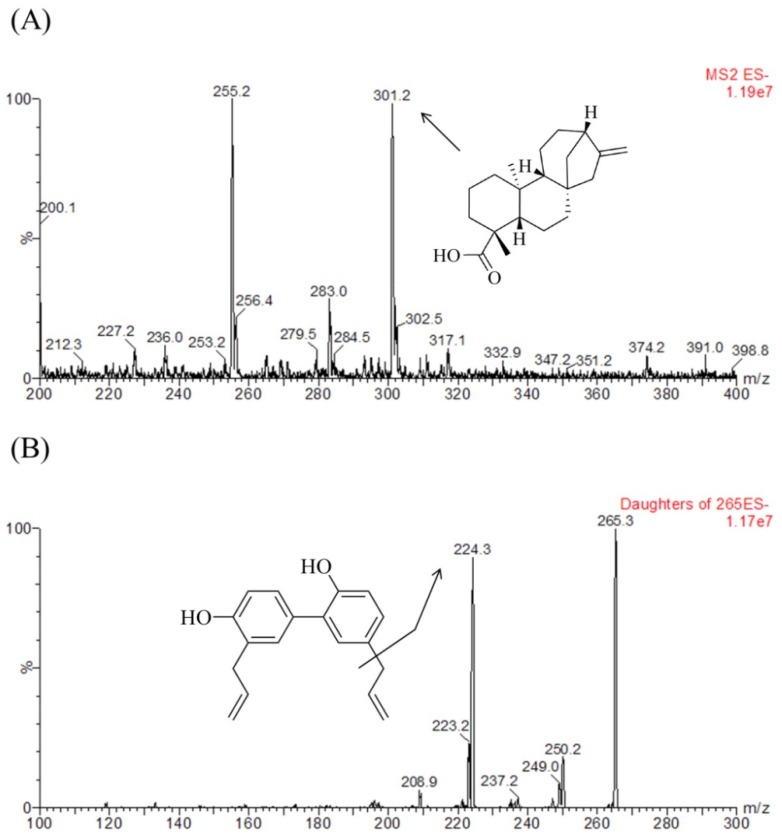
Negative ion electrospray mass scan spectra of (**A**) KAU and (**B**) honokiol (IS).

Chromatographic condition, sample preparation method, and mass spectrometric parameters such as the capillary voltage, collision energy, desolvation temperature, ion source temperature, and flow rate of desolvation and cone gases were optimized for the determination of KAU and IS. Figure 2 shows full scan product mass spectra of KAU and IS.

**Figure 3:**
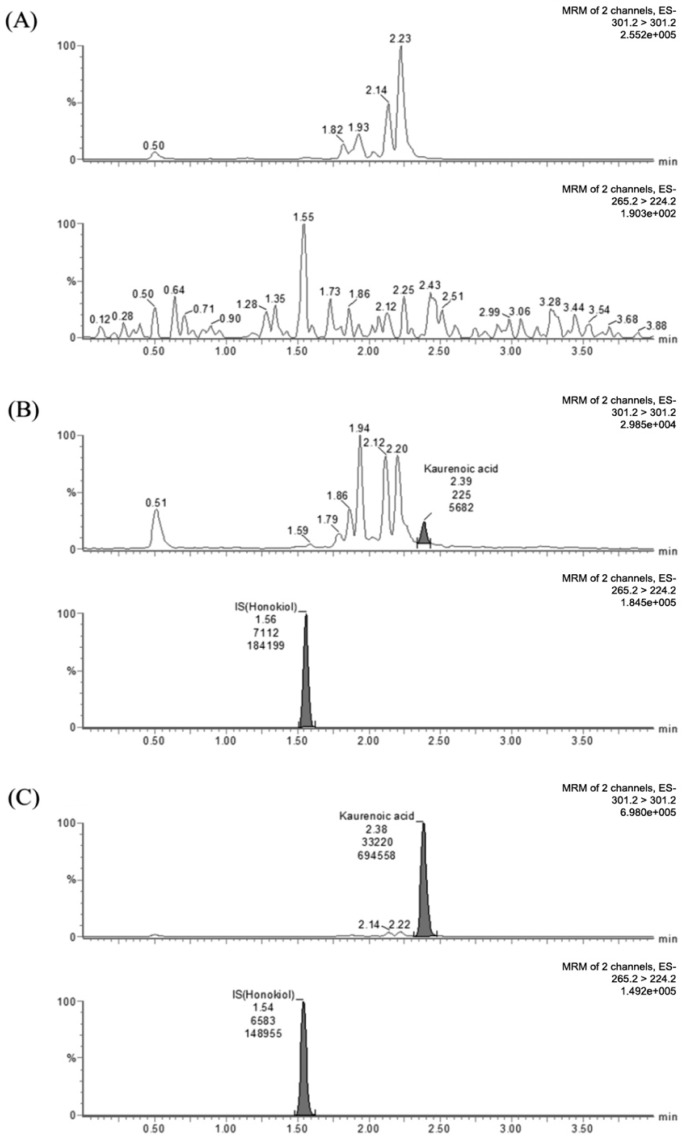
Representative Multiple reaction monitoring (MRM) chromatograms of KAU in human plasma samples. (**A**) Blank human plasma extract, (**B**) Human plasma sample extract containing KAU at LLOQ of 0.2 ng/mL and IS at 10 ng/mL, (**C**) human plasma sample extract (15.32 ng/mL for KAU) taken at 1 h after clinical oral dose administration of Araliae continentalis Radix extract powder (1.0054 g containing 1.147 mg KAU).

MRM transitions for KAU and IS were at m/z 301.2→301.2 and 265.3→224.3, respectively. (The *m/z* −255.2 was to unknown peak in the blank matrix and no interference to detect KAU with optimizing MRM.). For KAU, the intensity of daughter ion was extremely low and the intensity of parent ion was sufficiently detected. For this reason, we used pseudo MRM (*m/z* 301.2→301.2) and Gasparetto et al. [25] also reported that they were used *m/z* 301.1 for the detection of KAU to estimate PK profile of KAU. Generally for pseudo MRM collision energy used is kept low in order to achieve better abundance of parent peak. We also examined the various collision energies to obtain the best abundance of parent peak. When we tested at 30 eV of collision energy, we got the highest sensitivity of KAU, therefore, the 30 eV was chosen as collision energy for KAU. In addition, we could not find Na adduct with our developed analytical method although there were some reported papers that they detected Na adduct in detecting any compound not KAU. Considering the selectivity and effects of co-eluting peak for KAU, we optimized the LC chromatography method. The selectivity for KAU could be confirmed through Figure 3A,B, and it was suitable in accordance with the FDA guidance criteria [29]. The retention time of KAU was 2.39 min and that of IS was 1.56 min (Figure 3).

First, we investigated various mobile phases A such as water and variable acidic and buffer solutions including formic acid, acetic acid, ammonium formate, and ammonium acetate to find the most pertinent mobile phase for analysis of KAU. The peak of KAU was not detected when water was used as mobile phase A. In contrast, the peak area of KAU was significantly increased when acidic and buffer mobile phase such as water containing formic acid, acetic acid, and ammonium formate or ammonium acetate was used. Among these candidates, when 5 mM ammonium acetate in water was used as mobile phase A, the intensity was higher than others and proper peak shape, retention time, and interference peaks were observed.

We also compared protein precipitation using methanol and acetonitrile by LLE using ethyl acetate, ethyl ether, methyl-*tert*-butyl ether, and methylene chloride to determine the best sample preparation method. According to results of each method, the extraction using methyl-*tert*-butyl ether was better than other preparation methods for intensity at a low concentration. For this reason, the LLE method using methyl-*tert*-butyl ether was found to be the optimum preparation method for the determination of KAU in human plasma.

### 3.3. Quantitative Method Validation

Method validation was conducted in accordance with the FDA guidance [29]. All results ranged within the criteria of guidance. Thus, the developed bioanalytical method for quantification of KAU was successfully validated.

The selectivity was confirmed by comparing chromatograms of blank plasma sample, blank plasma containing KAU at LLOQ, and human plasma sample after oral administration. All peaks of KAU and IS separated with high resolution. No chromatographic interference from endogenous substances was observed at retention time of KAU or IS.

The calibration curve for KAU in human plasma exhibited a good linearity over the concentration range of 0.2–100 ng/mL for KAU, with correlation coefficients exceeding 0.997 at each batch (n = 5). Typical linear regression equation of the calibration curve was y = (0.15222 ± 0.01135)x + (0.00614 ± 0.00122) for KAU by plotting peak area ratio (y) of KAU to IS versus nominal concentration (x) of KAU that was weighted [9]. The developed UPLC-MS/MS analysis in our study provided LLOQ that was sufficient for further PK study after oral administration of Araliae continentalis Radix extract powder in humans.

Results of intra- and inter-batch precision and accuracy, as well as the stability of KAU under various conditions (n = 5), are summarized in Table 1 and Table 2.

CVs of precision were within 10.55% for QC samples. Results of intra- and inter-batch accuracy ranged from 94.07 to 102.90%. Detected concentrations for five replicates at low and high concentrations deviated within ±14.65%, demonstrating that KAU was stable in human plasma at room temperature for 8 h, at −80 °C for 3 months, after three freeze and thaw cycles, and at 10 °C in an autosampler for 6 h after the preparation procedure.

Extraction recovery of KAU from human plasma was 95.1 ± 5.4% while the recovery of IS was 93.4 ± 9.8%. Thus, this simple LLE method was successfully applied to the determination of KAU in human plasma after clinical oral dose administration of Araliae continentalis Radix extract powder.

### 3.4.Clinical Pharmacokinetic Study

Demographic characteristics, biomarkers related renal and hepatic function, and lipid blood tests data of the 10 subjects participated in this clinical study are summarized in Table 3.

In the present study, we identified characteristics of PKs of KAU in humans for the first time. Compatibilities of each PK model based on AIC, condition numbers, and count of minimization iteration were compared to determine the best optimized PK model. One- and two-compartment PK model with/without lag time were applied to describe the observed PK data. The model of two-compartment with first-order absorption was found to be the best optimized PK model due to its significantly low AIC, condition numbers, and count of minimization iteration compared to other models (data not shown).

**Figure 4:**
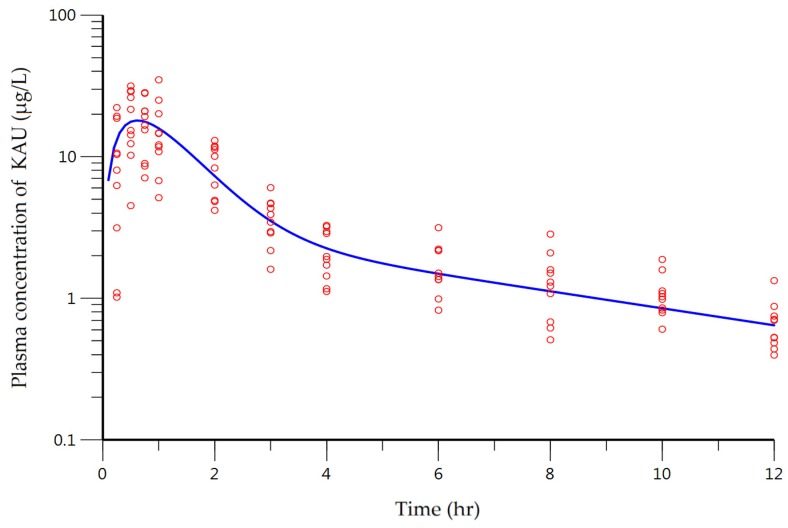
Plasma concentration-time profile described by the two-compartment model of KAU after oral administration of Araliae continentalis Radix extract powder (1.0054 g containing as 1.147 mg KAU) to human subjects (n = 10). Open circles and solid line represent observed and model predicted plasma concentrations of KAU, respectively.

The plasma concentration–time curve of KAU described by the two-compartment model with first-order absorption without lag time is illustrated in Figure 4. The predicted line well captured the trend of the observed data.

**Figure 5:**
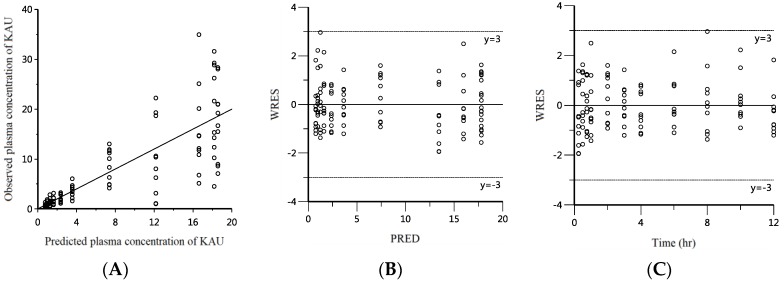
Diagnostic plots for KAU. (**A**) Observations plotted against model predicted plasma concentration. Weighted residuals against model predictions (**B**) or time (**C**).

Diagnostic plots shown in Figure 5 contained predicted plasma concentration of KAU (PRED) versus observed plasma concentration plot (A). There was a good agreement between observed concentration and PRED. In plots of weighted residuals versus PRED (B) or time (C), there was no obvious trend. Residuals were symmetrically distributed around zero.

Estimated results of PK parameters were: 23.89 L/h for CL/F, 15.55 L/h for CL_D_/F, 24.44 L for V/F, 64.05 L for V_2_/F, and 1.72 h^−1^ for k_a_. Coefficient of variation (CV) of PK parameters was approximately within 20% with appropriate degree of precision (Table 4). However, two PK parameters had relatively large CV%: V/F and k_a_. The total volume of distribution was 88.49 L. Compared to 3 L, the volume of human plasma [35], KAU was well distributed throughout the body. In terms of physicochemical property of KAU, the calculated LogP was 5.04 (ChemDraw Ultra^®^ ver. 7.0.1, CambridgeSoft Corporation, Waltham, MA, USA). This means that KAU has high lipophilicity which indicates that the hydrophobic interaction between biological membrane and KAU can play an important role in the absorption of KAU. According to results of Caco-2 bidirectional transport assay, P_app_ was increased in a concentration-dependent manner with a linear pattern between concentration of KAU and P_app_ of AP to BL despite the action of the efflux transporters. Among demographic data, body weight and height ranged from 60.4 to 86.7 kg and from 157.0 to 183.0 cm, respectively. These factors can lead to variations in the volume of distribution of KAU.

The other PK parameter with relatively large CV was rate constant for absorption. Its estimated value was 1.72 h^−1^ with CV of 28.79%. The cause of this variability might be primarily in terms of the sparse data on the absorption phase. In addition, there was inter-individual variability (IIV) in the physiological absorption process. In the observed versus PRED plot as shown in Figure 5A, in the low concentration range of 0 to 4 μg/L, observation value and predicted value were well superimposed. However, when the concentration was increased, the distribution between observation and PRED was also increased. This phenomenon can be interpreted by applying proportional error model. The visually determined C_max_ from individual time-plasma concentration profile ranged from 10.24 to 34.29 μg/L. This is another example of significant variability in the absorption process. The cause of this IIV can occur via transporters involved in the absorption process. Thus, we investigated the role of efflux transporters in the absorption of KAU.

To clarify the underlying cause of the IIV of parameters, non-linear mixed effect modeling was conducted. After establishing the basic model, we explored potential covariates, such as age, body weight, height, SCr, CrCl, ALT, AST, and GGTP etc., to explain IIV of primary parameters. In addition, we considered the relationship could be explained by physiologically plausible. Following this process, we only identified the relationship between CrCl and CL/F. However, even after incorporation of the CrCl as covariate, the basic model was not significantly improved. Therefore in this study, the PK parameters derived from the basic model were presented. It might be due to the limitations of this study that are the PK data derived from on ethnic group (healthy male Koreans) and small sample size (only 10 subjects participated in this trial). According to the Savic and Karlsson, the quality of the individual parameter estimate heavily depends on the observed data available [36]. As a further study, the pharmacogenetics of biochemical factors which might affect the IIV of PK parameters of KAU would be additionally investigated.

### 3.5. Cytotoxicity Assay of KAU

**Figure 6:**
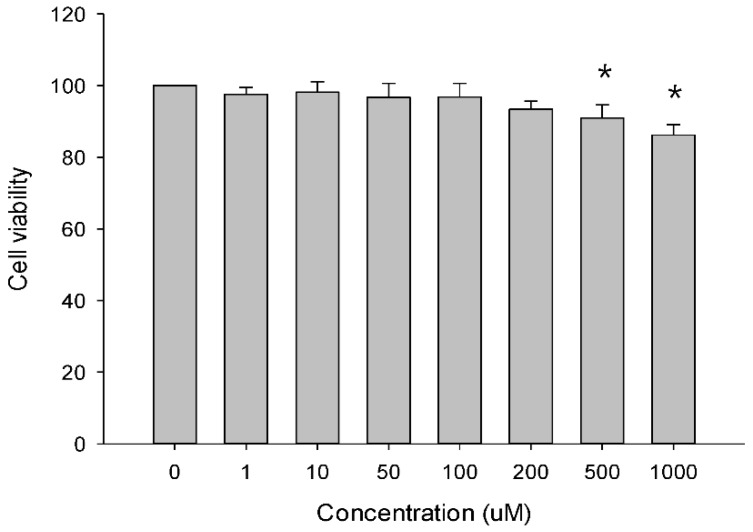
Cytotoxicity of KAU in Caco-2 cells by CCK-8 assay (mean ± SD, n = 6). * *p* < 0.05.

Cytotoxicity of KAU treatment at different concentrations (1, 10, 50, 100, 500, and 1000 μM) for 24 h to Caco-2 cells was evaluated by CCK-8 assay. As shown in Figure 6, treatment with KAU at 1, 10, 50, or 100 μM for 24 h had no cytotoxic effects on Caco-2 cells. Therefore, these concentration levels were chosen as concentrations of KAU for bidirectional transport assay.

### 3.6. Bidirectional Transport Assay

Based on results of cytotoxicity analysis, bidirectional membrane transport of KAU at concentrations of 1, 10, 50, and 100 µM in Caco-2 cell monolayer was evaluated. KAU was stable after 2 h of incubation in the transport buffer at 37 °C.

According to FDA guidance [34] and Volpe DA et al. [32], it was reported that P_app_ of propranolol and nadolol as high and low permeability drug were 30.76 ± 1.91 and 0.62 ± 0.18 × 10^−6^ cm/s, respectively. In this study, the permeability of KAU increased with concentration-dependent manner. So it is hard to compare directly with these values, but we could qualitatively interpret the relationship between the C_max_ of KAU and P_app__(AP→BL__)_. The estimated C_max_ of KAU was 18.02 μg/L, and approximately 60 μM, and calculated P_app__(AP→BL__)_ by linear regression line based on the result in the Table 5 at C_max_ was 28.00 × 10^−6^ cm/s. Therefore, it is suggested that KAU is highly permeable through intestinal membrane by comparing with permeability of propranolol. 

As shown in Table 5, KAU transport was higher in secretory (BL→AP) direction than that in absorptive (AP→BL) direction. In FDA guidance [37], any compound with observed efflux ratio (ER) of 2 or more is considered a potential substrate of active efflux transporters. ER of KAU was 1.70 to 2.37 (Table 5). Thus, it was uncertain whether KAU was a substrate of efflux transporters. Liang et al. [38] have reported the transport of analytes through inhibition study, although their efflux ratios were less than 2. Sun et al. [39] has also reported a passive diffusion and transport by efflux transporters as an absorption mechanism of analytes. In addition, the P_app(AP__→__BL)_ of KAU was increased in a concentration-dependent manner. If the efflux transport is saturated, the efflux rate is fixed as constant. So this phenomenon could be explained by saturating absorption process. Therefore, we suggest that KAU is a potential substrate of efflux transporter and an additional inhibition study needs to be performed to confirm the clear absorption mechanism of KAU in the future.

## 4. Conclusions

In this study, an analytical method using UPLC-MS/MS was developed and validated for KAU in human plasma. This is the first study to evaluate PKs of KAU in humans and investigate the mechanism of its absorption. Based on our results, we will perform population pharmacokinetic analysis to explain the IIV of PK parameters (i.e., clearance, volume of distribution, and absorption rate constant etc.) with additional clinical trials and reveal metabolic characteristics and elimination mechanism of KAU to explain the relationship between PK parameters. Potential covariates affecting the variation need to be explored in the future. In addition, we need to identify which transporters involved in the absorption process of KAU to clarify PK properties of KAU in future studies.

## Figures and Tables

**Table 1:** Precision and accuracy of UPLC-MS/MS analysis for the determination of KAU in human plasma.

Spiked (ng/mL)	Measured (Mean ± SD)	Precision (CV, %)	Accuracy (%)
Intra-batch (n = 5)			
0.2	0.195 ± 0.013	6.72	97.50
0.6	0.564 ± 0.021	3.67	94.07
16	16.052 ± 0.182	1.13	100.33
80	79.783 ± 5.366	6.73	99.73
Inter-batch (n = 5)			
0.2	0.206 ± 0.022	10.55	102.90
0.6	0.577 ± 0.034	5.91	96.21
16	15.958 ± 0.558	3.49	99.74
80	78.082 ± 2.114	2.71	97.60

**Table 2:** Stability of KAU under various conditions.

Condition	Spiked Concentration (ng/mL)	Measured Concentration (Mean ± SD)	Deviation (%)
Freeze and thaw stability (3 cycles)	0.6	0.54 ± 0.02	−4.72
	80	75.41 ± 2.15	−6.15
Short-term stability (25 °C for 8 h)	0.6	0.54 ± 0.05	−3.83
	80	75.08 ± 0.77	−5.89
Long-term stability (−80 °C for 3 months)	0.6	0.48 ± 0.03	−14.65
	80	69.20 ± 7.78	−13.27
Post-preparative stability (10 °C for 6 h)	0.6	0.51 ± 0.04	−9.21
	80	71.46 ± 2.45	−10.44

**Table 3:** Characteristics of 10 healthy Korean male subjects.

Characteristic (Units)	Mean	SD	Median	Range
Age (years)	25.8	4.3	24.5	21.0–33.0
Body weight (kg)	73.3	6.8	73.65	60.4–86.7
Height (cm)	172.5	6.5	173.5	157.0–183.0
SCr (mg/dL)	0.95	0.09	0.97	0.80–1.10
CrCl (mL/min)	115.0	11.8	115.7	98.0–139.1
BUN (mg/dL)	17.4	11.7	14.3	10.3–51.9
Total protein (g/dL)	8.0	0.2	8.1	7.6–8.3
Total cholesterol (mg/dL)	170.2	16.6	167.6	148.1–203.4
Triglyceride (mg/dL)	148.3	31.0	139.2	95.7–201.0
Albumin (g/dL)	5.01	0.12	4.98	4.83–5.20
Alk (U/L)	65.6	14.5	64.8	47.3–93
ALT (U/L)	20.2	5.0	19.5	12.3–32.1
AST (U/L)	24.7	6.0	24.5	16.8–39.0
GGTP (U/L)	21.4	5.0	20.2	15.2–34.4

**Table 4:** Model estimated pharmacokinetic (PK) parameters of KAU.

Parameter	Unit	Definition	Estimate	CV%
Primary parameters				
CL/F	L/h	Systemic clearance	23.89	9.30
CL_D_/F	L/h	Inter-compartmental clearance	15.55	13.77
K_a_	h^−1^	Rate constant for absorption	1.72	28.79
V/F	L	Volume of distribution of the central compartment	24.44	29.16
V_2_/F	L	Volume of distribution of the peripheral compartment	64.05	21.05
ε	-	Proportional residual error	0.48	11.09
Secondary parameters *				
C_max_	μg/L	Peak plasma concentration	18.02	-
t_max_	h	Time to reach the peak plasma concentration	0.6	-
AUC	μg∙h/L	Area under the time-plasma curve	47.89	-
t_1/2_	h	Plasma elimination half-life	4.97	-

* Secondary parameters were estimated by non-compartmental analysis with data of predicted plasma concentration of KAU.

**Table 5:** Permeability of KAU in Caco-2 cell monolayer (mean ± SD, n = 6).

KAU (µM)	Caco-2 P_app_ (× 10^−6^ cm/s)		Efflux Ratio
	AP→BL	BL→AP	
1	0.18 ± 0.03	0.34 ± 0.01	1.98
10	4.47 ± 0.64	7.40 ± 1.55	1.70
50	21.78 ± 5.93	44.3 ± 2.71	2.15
100	49.42 ± 18.38	107.26 ± 32.14	2.37

## References

[1] Lim H., Jung H.A., Choi J.S., Kim Y.S., Kang S.S., Kim H.P. (2009). Anti-inflammatory activity of the constituents of the roots of aralia continentalis. Arch. Pharm. Res..

[2] Park H.J., Hong M.S., Lee J.S., Leem K.H., Kim C.J., Kim J.W., Lim S. (2005). Effects of aralia continentalis on hyperalgesia with peripheral inflammation. Phytother. Res..

[3] Cho S.O., Ban J.Y., Kim J.Y., Jeong H.Y., Lee I.S., Song K.-S., Bae K., Seong Y.H. (2009). Aralia cordata protects against amyloid β protein (25–35)–induced neurotoxicity in cultured neurons and has antidementia activities in mice. J. Pharmacol. Sci..

[4] Liu X., Hou D., Zhao N., Wang B. (2010). Extraction and antioxidant activity of flavonoids from aralia cordata. J. Chin. Med. Mater..

[5] Cheng W.L., Lin T.Y., Tseng Y.H., Chu F.H., Chueh P.J., Kuo Y.H., Wang S.Y. (2011). Inhibitory effect of human breast cancer cell proliferation via p21-mediated g1 cell cycle arrest by araliadiol isolated from aralia cordata thunb. Planta Med..

[6] Kim T.D., Lee J.Y., Cho B.J., Park T.W., Kim C.J. (2010). The analgesic and anti-inflammatory effects of 7-oxosandaracopimaric acid isolated from the roots of aralia cordata. Arch. Pharm. Res..

[7] Kang S. (1997). Chemistry and biological activity of the constituents from aralia species. Ann. Rep. Nat. Prod. Sci..

[8] Okoye T.C., Akah P.A., Omeje E.O., Okoye F.B., Nworu C.S. (2013). Anticonvulsant effect of kaurenoic acid isolated from the root bark of Annona senegalensis. Pharmacol. Biochem. Behav..

[9] Lizarte Neto F.S., Tirapelli D.P.C., Ambrosio S.R., Tirapelli C.R., Oliveira F.M., Novais P.C., Peria F.M., Oliveira H.F., Carlotti Junior C.G., Tirapelli L.F. (2013). Kaurene diterpene induces apoptosis in u87 human malignant glioblastoma cells by suppression of anti-apoptotic signals and activation of cysteine proteases. Braz. J. Med. Biol. Res..

[10] Cavalcanti B.C., Bezerra D.P., Magalhaes H.I., Moraes M.O., Lima M.A.S., Silveira E.R., Camara C.A., Rao V.S., Pessoa C., Costa-Lotufo L.V. (2009). Kauren-19-oic acid induces DNA damage followed by apoptosis in human leukemia cells. J. Appl. Toxicol..

[11] Velikova M., Bankova V., Tsvetkova I., Kujumgiev A., Marcucci M.C. (2000). Antibacterial ent-kaurene from brazilian propolis of native stingless bees. Fitoterapia.

[12] Cotoras M., Folch C., Mendoza L. (2004). Characterization of the antifungal activity on botrytis cinerea of the natural diterpenoids kaurenoic acid and 3β-hydroxy-kaurenoic acid. J. Agric. Food Chem..

[13] Santos A.O., Izumi E., Ueda-Nakamura T., Dias-Filho B.P., Veiga-Junior V.F., Nakamura C.V. (2013). Antileishmanial activity of diterpene acids in copaiba oil. Mem. Inst. Oswaldo Cruz.

[14] Batista R., Garcia P.A., Castro M.A., Miguel Del Corral J.M., Speziali N.L., de P Varotti F., de Paula R.C., Garcia-Fernandez L.F., Francesch A., San Feliciano A. (2013). Synthesis, cytotoxicity and antiplasmodial activity of novel ent-kaurane derivatives. Eur. J. Med. Chem..

[15] Pereira S., Taleb-Contini S., Coppede J., Pereira P., Bertoni B., Franca S., Pereira A.M. (2012). An ent-kaurane-type diterpene in croton antisyphiliticus mart. Molecules.

[16] De Andrade B.B., Moreira M.R., Ambrosio S.R., Furtado N., Cunha W.R., Heleno V., Silva A.N., Simao M.R., Da Rocha E., Martins C. (2011). Evaluation of ent-kaurenoic acid derivatives for their anticariogenic activity. Nat. Prod. Commun..

[17] Vilegas J.H., de Marchi E., Lanças F.M. (1997). Determination of coumarin and kaurenoic acid in mikania glomerata (“guaco”) leaves by capillary gas chromatography. Phytochem. Anal..

[18] Costa-Lotufoa L.V., Cunha G.M., Fariasa P.A., Vianaa G.S., Cunhaa K.M., Pessoaa C., Moraes M.O., Silveirab E.R., Gramosab N.V., Rao V.S. (2002). The cytotoxic and embryotoxic effects of kaurenoic acid, a diterpene isolated from copaifera langsdorffii oleo-resin. Toxicon.

[19] Mizokami S.S., Arakawa N.S., Ambrosio S.R., Zarpelon A.C., Casagrande R., Cunha T.M., Ferreira S.H., Cunha F.Q., Verri W.A. (2012). Kaurenoic acid from sphagneticola trilobata inhibits inflammatory pain: Effect on cytokine production and activation of the no-cyclic gmp-protein kinase g-atp-sensitive potassium channel signaling pathway. J. Nat. Prod..

[20] Cavalcanti B.C., Ferreira J.R., Moura D.J., Rosa R.M., Furtado G.V., Burbano R.R., Silveira E.R., Lima M.A., Camara C.A., Saffi J. (2010). Structure-mutagenicity relationship of kaurenoic acid from xylopia sericeae (annonaceae). Mutat. Res..

[21] Guillope R., Escobar-Khondiker M., Guerineau V., Laprevote O., Hoglinger G.U., Champy P. (2011). Kaurenoic acid from pulp of annona cherimolia in regard to annonaceae-induced parkinsonism. Phytother. Res..

[22] Oliveira B.H., Sant’Ana A.E., Bastos D.Z. (2002). Determination of the diterpenoid, kaurenoic acid, in annona glabra by hplc. Phytochem. Anal..

[23] Smith D.A., Humphrey M.J., Charuel C. (1990). Design of toxicokinetic studies. Xenobiotica.

[24] Lan K., Xie G., Jia W. (2013). Towards polypharmacokinetics: Pharmacokinetics of multicomponent drugs and herbal medicines using a metabolomics approach. Evid.-Based Complement. Altern. Med..

[25] Gasparetto J.C., Peccinini R.G., de Francisco T.M., Cerqueira L.B., Campos F.R., Pontarolo R. (2015). A kinetic study of the main guaco metabolites using syrup formulation and the identification of an alternative route of coumarin metabolism in humans. PLoS ONE.

[26] Matos D.M., Viana M.R., Alvim M.C.O., Carvalho L.S.A., Leite L.H.R., Da Silva Filho A.A., Nascimento J.W.L. (2018). Pharmacokinetic profile and oral bioavailability of kaurenoic acid from *Copaifera* spp. In rats. Fitoterapia.

[27] Leslie E.M., Deeley R.G., Cole S.P. (2005). Multidrug resistance proteins: Role of p-glycoprotein, mrp1, mrp2, and bcrp (abcg2) in tissue defense. Toxicol. Appl. Pharmacol..

[28] Madgula V.L., Avula B., Choi Y.W., Pullela S.V., Khan I.A., Walker L.A., Khan S.I. (2008). Transport of schisandra chinensis extract and its biologically-active constituents across caco-2 cell monolayers—An in-vitro model of intestinal transport. J. Pharm. Pharmacol..

[29] FDA (2013). The Guidance for Industry: Bioanalytical Method Validation.

[30] FDA (2016). Botanical Drug Development Guidance for Industry.

[31] Jung H.R., Kim S.J., Ham S.H., Cho J.H., Lee Y.B., Cho H.Y. (2014). Simultaneous determination of puerarin and its active metabolite in human plasma by uplc-ms/ms: Application to a pharmacokinetic study. J. Chromatogr. B.

[32] Donna A.V., Patrick J.F., Anthony B.C., Ebenezer B.A., Christopher D.E., Yu L.X., Ajaz S.H. (2007). Classification of drug permeability with a caco-2 cell monolayer assay. Clin. Res. Regul. Aff..

[33] Hubatsch I., Ragnarsson E.G., Artursson P. (2007). Determination of drug permeability and prediction of drug absorption in caco-2 monolayers. Nat. Protoc..

[34] FDA (2017). Waiver of In Vivo Bioavailability and Bioequivalence Studies for Immediate-Release Solid Oral Dosage forms Based on a Biopharmaceutics Classification System Guidance for Industry.

[35] Davies B., Morris T. (1993). Physiological parameters in laboratory animals and humans. Pharm. Res..

[36] Savic R.M., Karlsson M.O. (2009). Importance of shrinkage in empirical bayes estimates for diagnostics: Problems and solutions. AAPS J..

[37] FDA (2012). Guidance for Industry: Drug Interaction Studies.

[38] Liang X.L., Zhao L.J., Liao Z.G., Zhao G.W., Zhang J., Chao Y.C., Yang M., Yin R.L. (2012). Transport properties of puerarin and effect of radix angelicae dahuricae extract on the transport of puerarin in caco-2 cell model. J. Ethnopharmacol..

[39] Sun S., Zhang H., Sun F., Zhao L., Zhong Y., Chai Y., Zhang G. (2014). Intestinal transport of sophocarpine across the caco-2 cell monolayer model and quantification by lc/ms. Biomed. Chromatogr..

